# Delayed development induced by toxicity to the host can be inherited by a bacterial-dependent, transgenerational effect

**DOI:** 10.3389/fgene.2014.00027

**Published:** 2014-02-25

**Authors:** Yael Fridmann-Sirkis, Shay Stern, Michael Elgart, Matana Galili, Amit Zeisel, Noam Shental, Yoav Soen

**Affiliations:** ^1^Department of Biological Chemistry, Weizmann Institute of ScienceRehovot, Israel; ^2^Department of Physics of Complex Systems, Weizmann Institute of ScienceRehovot, Israel; ^3^Department of Computer Science, The Open UniversityRaanana, Israel

**Keywords:** host–microbe interactions, non-Mendelian inheritance, *D. melanogaster*, epigenetics, stress responses

## Abstract

Commensal gut bacteria in many species including flies are integral part of their host, and are known to influence its development and homeostasis within generation. Here we report an unexpected impact of host–microbe interactions, which mediates multi-generational, non-Mendelian inheritance of a stress-induced phenotype. We have previously shown that exposure of fly larvae to G418 antibiotic induces transgenerationally heritable phenotypes, including a delay in larval development, gene induction in the gut and morphological changes. We now show that G418 selectively depletes commensal *Acetobacter* species and that this depletion explains the heritable delay, but not the inheritance of the other phenotypes. Notably, the inheritance of the delay was mediated by a surprising trans-generational effect. Specifically, bacterial removal from F1 embryos did not induce significant delay in F1 larvae, but nonetheless led to a considerable delay in F2. This effect maintains a delay induced by bacterial-independent G418 toxicity to the host. In line with these findings, reintroduction of isolated *Acetobacter* species prevented the inheritance of the delay. We further show that this prevention is partly mediated by vitamin B2 (Riboflavin) produced by these bacteria; exogenous Riboflavin led to partial prevention and inhibition of Riboflavin synthesis compromised the ability of the bacteria to prevent the inheritance. These results identify host–microbe interactions as a hitherto unrecognized factor capable of mediating non-Mendelian inheritance of a stress-induced phenotype.

## Introduction

Transgenerational epigenetic phenomena have been reported in a number of different animal and plant species (Cavalli and Paro, 1998; Morgan et al., 1999; Kaati et al., 2002; Sollars et al., 2003; Anway et al., 2005; Cropley et al., 2006; Molinier et al., 2006; Lumey et al., 2007; Xing et al., 2007; Greer et al., 2011; Rechavi et al., 2011; Seong et al., 2011; Crews et al., 2012; Stern et al., 2012). These events may be induced by the environment, and have been shown to involve a variety of host-intrinsic factors and molecular pathways (Jablonka and Raz, 2009), including chromatin modifiers (Cavalli and Paro, 1998; Greer et al., 2011; Seong et al., 2011), DNA methylation (Xing et al., 2007; Heijmans et al., 2008; Carone et al., 2010; Guerrero-Bosagna et al., 2010; Schmitz et al., 2011), RNAi machinery (Rechavi et al., 2011; Ashe et al., 2012; Buckley et al., 2012), and secreted signals (McCaffery et al., 1998). Involvement of other host intrinsic or extrinsic factors is also possible but has been largely overlooked. Of these, the commensal gut microbiome is a particularly attractive candidate. The gut microbiome is an integral part of the development and homeostasis of its host (Rosenberg and Zilber-Rosenberg, 2011; Charroux and Royet, 2012; Buchon et al., 2013), but nonetheless, can be extensively modified by the environment. As with other environmental perturbations, disruption of the gut microbiome may lead to changes in the host which extend beyond one generation. However, potential multigenerational impacts of commensal gut bacteria on host development and physiology have not yet been investigated because typical studies assume that these bacteria affect each generation in a similar way.

Here, we examine multigenerational impacts of microbial changes using a model based on exposure of fly larvae to G418 toxicity in specific tissues. We have recently shown that this exposure can induce developmental phenotypes which persist for multiple generations in non-exposed offspring (Stern et al., 2012). In this system, we supplemented the larval food with G418 antibiotic and placed a resistance transgene fused to *GFP* (*neoGFP*) under the regulation of arbitrary, spatio-temporally restricted developmental promoters. This leads to toxic stress in tissues that are exposed to G418 but do not express sufficient levels of the “rescue,” *neoGFP* gene. Exposure to G418 led to multiple phenotypes, including a delay in larval development, promoter-dependent induction of *neoGFP* expression and morphological changes in two promoter cases. Moreover, some of the induced phenotypes persisted in a number of subsequent generations of non-exposed offspring (Stern et al., 2012). In particular, the delay in development and the induction of *neoGFP* expression were inherited at high penetrance and typically persisted for 3–10 generations without G418. One of the morphological phenotypes, wing abnormalities in the *Hsp70::neoGFP* case, was also heritable albeit at a much lower penetrance.

As has been previously shown, exposure of flies to an antibiotic (Chlortetracycline) can have a direct influence on the host tissue as well as an indirect effect mediated by an impact of the antibiotic on the commensal microbiome (Ridley et al., 2013). This rationale also applies to G418 which is an aminoglycoside which blocks polypeptide synthesis in both eukaryotic and prokaryotic cells. Thus, the above paradigm of G418-induced inheritance may provide a model for investigating potential contributions of host–microbe interactions to the inheritance of induced phenotypes in the host. We therefore, investigated the impact of G418 on the microbial composition and the resulting implications for induction and inheritance of responses in the host. We show that G418 leads to a selective depletion of commensal *Acetobacter* species. Removal of extracellular bacteria without exposure to G418 had an almost negligible effect on the first generation of bacterial-depleted larvae (F1), but nonetheless caused a considerable delay in larval development in the following generation (F2). The delay in offspring development following parental removal of gut bacteria was completely eliminated by re-introduction of a commensal *Acetobacter* species. Reintroduction of a commensal *Acetobacter* species also prevented the inheritance of the delay in development in offspring of G418-exposed flies. We further show that this prevention of the heritable delay is mediated in part by Riboflavin produced by the *Acetobacter* species.

These results show that environmental disruption of the gut microbiome can induce different effects in parents and offspring. They also uncover an unexpected scenario by which host–microbe interactions mediate the inheritance of delayed development in response to G418, namely: the delay in the parental generation is induced by a direct effect of G418 on the host tissue, but is maintained in non-exposed offspring by the transgenerational effect of *Acetobacter* depletion. Specifically, the *Acetobacter* depletion causes a modification in the parents which becomes phenotypic (delayed development) only in the offspring. We show that this transgenerational effect is responsible for the inheritance of the delay in development, but not for the inheritance of induced *neoGFP* expression and the inheritance of morphological changes.

The inter-generational difference between the rate of development in bacterial-depleted parents and offspring suggests that changes in the gut microbiome may influence the germline in the parents.

## Results

### G418 selectively depletes *acetobacter* species from the larval gut

**Figure 1:**
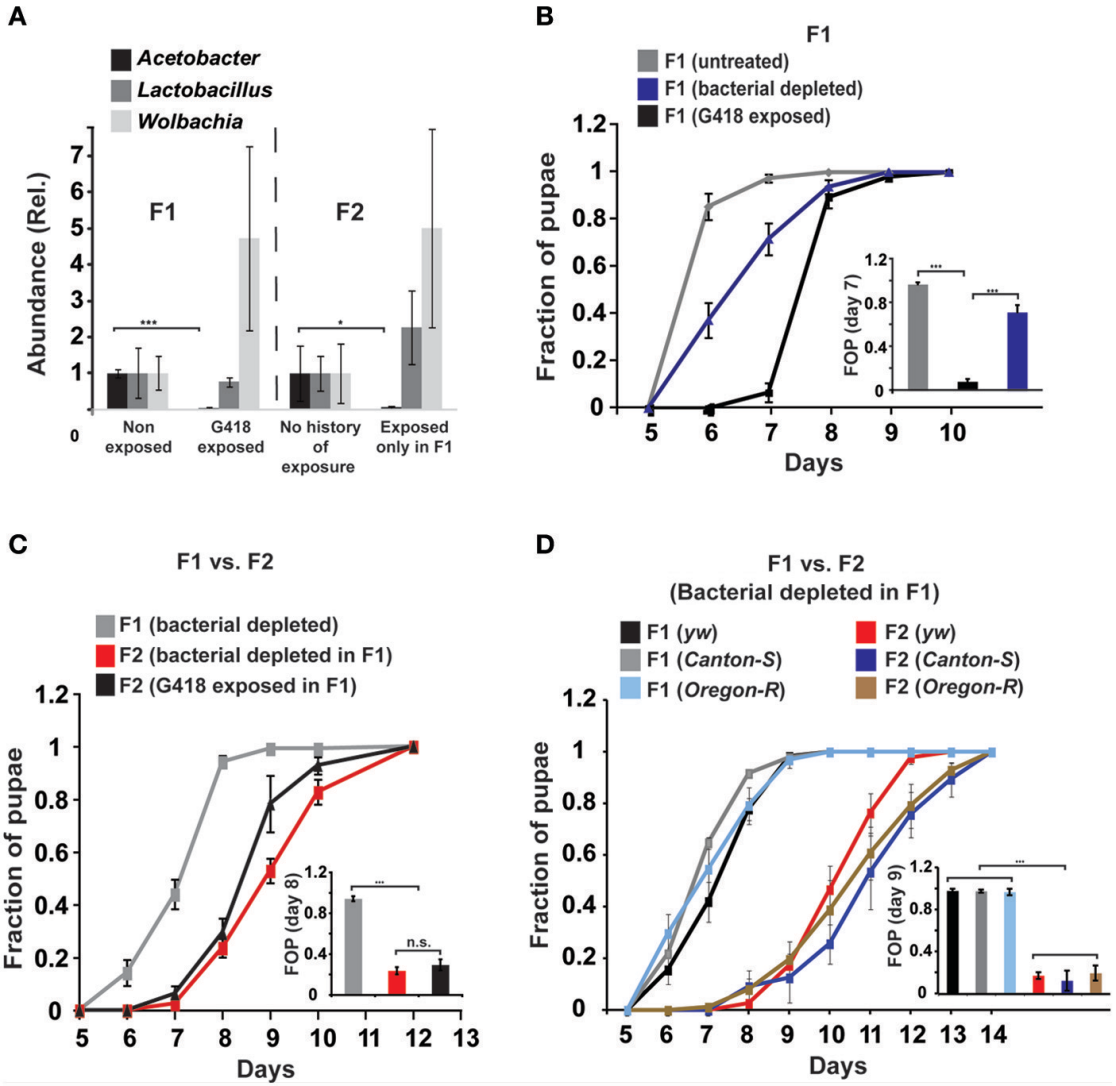
**Depletion of gut bacteria leads to a substantial delay in the development of the offspring, but not in the parental generation. (A)** Selective and heritable depletion of *Acetobacter* spp. following exposure to G418. Shown are measurements corresponding to two consecutive generations. Abundance of each bacterial type is normalized to the measured abundance in larvae without a history of G418 exposure. Mean fold-change ± SE of type-specific 16S DNA sequences extracted from guts of third instar larvae (*n* > 7). **(B)** Effect of egg dechorionation and sterilization (“Bacterial depleted”) on the kinetics of pupation of *hairy::neoGFP* larvae in the same generation. Note the small effect compared to the large delay following exposure to G418. Mean fraction of pupae ± SE in 7 vials. Inset: Statistical analysis of differences between fractions of pupae (FOP) in day 7. **(C)** Effect of egg dechorionation and sterilization in F1 on the kinetics of pupation in F1 and F2 *hairy::neoGFP* larvae. Note the large delay in F2 vs. F1 bacterial-depleted larvae, and the comparable kinetics of offspring of bacterial-depleted parents and offspring of G418-exposed parents. Inset: Statistical analysis of differences between fractions of pupae (FOP) in day 8. Mean fraction of pupae ± SE in at least three biological replicates. **(D)** Effect of egg dechorionation and sterilization in F1 on the kinetics of pupation in F1 and F2 in three non-transgenic fly lines (yw, Canton-S, and Oregon-R). Inset: Statistical analysis of differences between fractions of pupae (FOP) in day 9. ^*^p < 0.05, ^***^p < 0.001 (Student's *t*-test).

As a starting point for investigating the potential involvement of the gut microbiome in the response to G418, we tested if exposure to G418 modifies the composition of bacteria in the larva. We first analyzed the larval gut microbiome using an improved method of deep-sequencing of DNA coding for 16S ribosomal RNA (Amir et al., 2014). In line with recent findings (Wong et al., 2011), we identified in the gut of *hairy::neoGFP* larvae various *Acetobacter* and *Lactobacillus* spp. (Supplementary Data Sheet 1—Figure S1A, Supplementary Data Sheet 2). In addition to these extracellular species, we detected high abundance of the endosymbiont *Wolbachia*, which is known to exist in many lines of *D. melanogaster* (Bourtzis et al., 1996; Dobson et al., 1999; Veneti et al., 2003; McGraw and O'Neill, 2004) and is capable of manipulating various reproductive features of its host (Werren, 1997; Starr and Cline, 2002; Ikeya et al., 2009). The deep-sequencing analysis revealed strong reduction in the relative amount of *Acetobacter* spp. in G418-exposed larvae (Supplementary Data Sheet 1—Figure S1A). To validate this depletion, we developed a quantitative PCR-based assay capable of selectively measuring the total contents of *Acetobacter*, *Lactobacilus*, and *Wolbachia* spp. (Supplementary Data Sheet 1—Figures S1B–D). Measurement of the amounts of these three types of bacteria confirmed that G418 selectively depletes *Acetobacter* species (Figure 1A). Notably, the depletion of *Acetobacter* species persisted in non-exposed F2 offspring of G418 exposed flies (Figure 1A), indicating that the change in bacterial composition is itself heritable. Thus, G418 toxicity modifies the fly's microbiome by selectively depleting commensal *Acetobacter* species.

### The delay in development is inherited by a bacterial-dependent, transgenerational effect

We investigated whether the depletion of commensal bacteria might be responsible for the G418-induced phenotypes. We began by testing if removal of bacteria by egg dechorionation and sterilization (Brummel et al., 2004) can reproduce the delay observed in G418-treated larvae (Stern et al., 2012). We used the qPCR and bacterial growth assays to verify complete removal of extracellular bacteria (Supplementary Data Sheet 1–Figures S1E,F) and analyzed the influence of bacterial removal on pupation time. As previously demonstrated (Shin et al., 2011; Storelli et al., 2011), the effect of bacterial removal on pupation time depended on the food quality (Supplementary Data Sheet 1—Figure S2). Under the food conditions we have previously used (Stern et al., 2012), removal of bacteria led to a mild delay, which was considerably smaller than the delay caused by exposure to G418 (~0.5 vs. 2–3 days, respectively; Figure 1B, Supplementary Data Sheet 1—Figure S3A). The lack of a considerable delay was consistent with previous work in which germ-free larvae developed in high quality diet (Storelli et al., 2011). Thus, depletion of extracellular bacteria did not reproduce the G418-induced delay in the first generation. Nevertheless, analysis of F2 *hairy::neoGFP* larval offspring of bacterial-depleted F1 parents revealed a very substantial delay, comparable to the delay observed in offspring of G418 exposed parents (Figure 1C). This effect of bacterial removal was also observed in three non-transgenic lines (Figure 1D). The difference between the rate of development in bacterial depleted parents and offspring depended on the diet, and was mostly evident within a particular range of diet quality (Supplementary Data Sheet 1—Figure S2). Within this range, removal of gut bacteria had a transgenerational effect that was not observed in the first generation of larvae lacking the bacteria. This transgenerational effect of bacterial depletion can account for the delay in development in non-exposed offspring of G418-exposed (and hence, *Acetobacter*-depleted) parents. As has been noted (Ridley et al., 2013), direct evidence can be obtained by antibiotic exposure of flies that had been depleted of their bacteria prior to the exposure. Applying this strategy in our system showed that removal of extracellular bacteria prior to G418 exposure led to significant reduction in the heritable delay (Supplementary Data Sheet 1—Figure S4A). This verifies the key contribution of bacterial depletion to the inheritance of delayed development in response to parental exposure to G418.

Other phenotypes reported by Stern et al., were largely unaffected by the mere depletion of bacteria. Removal of extracellular bacteria by egg dechorionation without exposure to G418 had no effect on the levels of *neoGFP* in the gut of the larvae (Supplementary Data Sheet 1—Figure S3B). Similarly, we did not observe any of the morphological influences of G418 exposure in bacterial depleted flies (Supplementary Data Sheet 1—Figures S3C,D). Genome-wide analysis of mRNA levels in the proventriculus of bacterial-depleted *hairy::neoGFP* larvae further revealed very small overlap with the response to G418 (only 8 and 6% of genes that were up- and down-regulated in G418 were similarly affected by bacterial removal; Supplementary Data Sheet 1—Figure S3E, Supplementary Data Sheet 3). As expected, the set of transcripts that were down regulated following dechorionation were enriched for genes involved in response to bacterium (*p* < 10^−4^; Supplementary Data Sheet 1—Figure S2F, Supplementary Data Sheet 4), e.g., *Attacin*, and *Defensin* (Supplementary Data Sheet 1—Figure S3J). The set of up-regulated transcripts, on the other hand, was strongly enriched with genes associated with phospholipase activity and polysaccharide metabolism (*p* < 10^−8^ and *p* < 10^−4^, respectively; Supplementary Data Sheet 1—Figure S3F, Supplementary Data Sheet 4). These categories were not enriched in the set of proventriculus genes which responded to G418 (Supplementary Data Sheet 1—Figure S3F, Supplementary Data Sheet 4). Moreover, unlike in G418-exposed larvae (Stern et al., 2012), the depletion of bacteria did not down-regulate Polycomb group (PcG) genes (Supplementary Data Sheet 1—Figures S3G,H) and did not induce the anti-detoxification, *GstD* genes (Supplementary Data Sheet 1—Figure S3I). Further analysis of offspring of bacterial-depleted flies revealed that the difference between parents and their offspring with respect to the delay in development is not observed with respect to the other heritable phenotypes. Specifically, depletion of bacteria in F1 did not lead to induction of *neoGFP* expression in the larval gut of the offspring (Supplementary Data Sheet 1—Figures S4B,C), or to wing deformations in *Hsp70::neoGFP* offspring of bacterial depleted parents (Supplementary Data Sheet 1—Figure S4D).

Altogether, this shows that the transgenerational effect of bacterial depletion can account for the inheritance of the delay in development, but not for the other transgenerationally heritable phenotypes of G418 exposure.

### The transgenerational inheritance of the delay in development is prevented by reintroduction of the depleted *acetobacter* species

**Figure 2:**
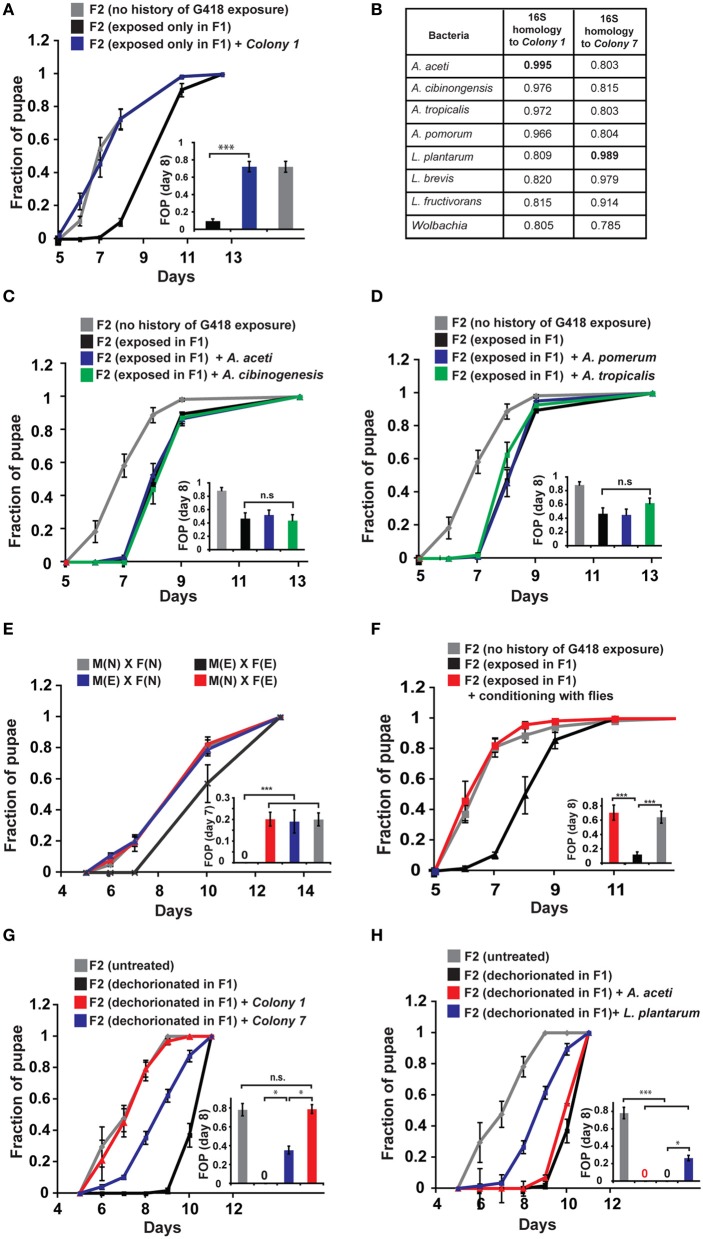
**Reintroduction of a commensal *Acetobacter* species prevents the inheritance of the delay in development. (A)** Supplementing the food with bacteria from a single *Acetobacter* colony (*Colony 1*) isolated from non-exposed flies, prevented the inheritance of the delay in non-exposed F2 offspring of G418-exposed parents. Mean fraction of pupae ± SE in 10 vials. All insets: Statistical analysis of differences between fractions of pupae (FOP) in the indicated day (7 or 8). **(B)** Analysis of 16S rRNA gene sequence of bacteria from two isolated colonies, *Colony 1* and *Colony 7*. Shown are computed similarities to various species of *Acetobacter, Lactobacillus*, and *Wolbachia*. **(C,D)** Supplementing fly vials with the non-commensal species, *A. aceti* or *A. cibinogenesis* **(C)**, and with *A. pomorum* or *A. tropicalis* **(D)**, did not prevent the inheritance of the delay. Mean fraction of pupae ± SE in 10 vials. **(E)** Kinetic curves of pupation of F2 larval offspring generated from crosses between G418-exposed and non-exposed *hairy::neoGFP* F1 males or virgin females (all four combinations are shown). In all cases, the F2 larvae were not exposed to G418. Delay in F2 was observed only when both parents were exposed to G418. Mean fraction of pupae ± SE in 6 vials. “M(E)”—exposed males “M(N)”—non-exposed males, “F(E)”—exposed females “F(N)”—non-exposed females. **(F)** Prevention of the inheritance of delayed development in vials that were temporarily exposed for 1 day (conditioning) to flies with no history of ancestral exposure to G418. Mean fraction of pupae ± SE in 5 vials. **(G)** Placing dechorionated and sterilized F1 eggs on food with bacteria from *Colony 1* prevented a delay in development of the F2 larvae. Bacteria from *Colony 7* led to only partial reduction in the delay. Mean fraction of pupae ± SE in at least 15 vials pooled from three biological replicates. **(H)** Same as **(G)** for food with *L. plantarum* from another stock (partial reduction) and non-commensal *A. aceti* (no effect). Mean fraction of pupae ± SE in at least 15 vials pooled from three biological replicates. ^*^p < 0.05, ^***^p < 0.001 (Student's *t*-test).

To test if reintroduction of depleted bacteria can prevent the inheritance of the delay in development following parental exposure to G418, we isolated bacterial colonies from non-exposed flies. We then tested the ability of bacteria from these colonies to prevent the inheritance in non-exposed offspring of G418 exposed (F1) *hairy::neoGFP* parents. Supplementing the fly food with bacteria from a single colony (*Colony 1*, Supplementary Data Sheet 1—Figure S5D) sufficed to prevent the inheritance of the delay in development (Figure 2A). Analysis of the 16S sequence of the ribosomal RNA gene of *Colony 1* revealed similarity to 4 *Acetobacter* spp. (Figure 2B): *A. aceti* (99.5% similarity to *Colony 1*), *A. cibinongensis* (97.6% similarity), *A. pomorum* (96.6% similarity), and *A. tropicalis* (97.2% similarity). However, the non-commensal *Acetobacter* spp., *A. aceti*, *A. pomorum*, *A. tropicalis* and *A. cibinongensis* (all obtained from the DSMZ stock collection) could not prevent the inheritance of the delay in development (Figures 2C,D). Thus, the prevention of the inheritance of the delay following parental exposure to G418 required commensal *Acetobacter* species.

Notably, prevention of the inheritance of the delay was also observed in various natural, mating-dependent, or independent settings, in which exposed flies were infected with bacteria carried by non-exposed flies. Specifically, when G418 exposed males or females were crossed to naïve partners (i.e., flies lacking any history of exposure), the offspring larvae were not delayed in their development (Figure 2E). Additionally, when G418-exposed parents were inserted into a vial which temporarily contained naïve flies (either males or virgin females) for 1 day prior to the insertion of the exposed parents, the offspring were no longer delayed (Figure 2F). Similar results were obtained when the vials were supplemented with PBS that was brought in contact with naïve flies, but not when this fluid was filtered to remove all the bacteria (Supplementary Data Sheet 1—Figure S5A). These findings show that the inheritance of the delay in development can be prevented by either natural or experimental exposures to bacteria from male or female flies with no history of exposure to G418.

While *Colony 1* supplementation always prevented the inheritance of the delay in offspring, it did not necessarily prevent the inheritance of induced *neoGFP* expression. Altogether, the average expression of *neoGFP* in the foregut of the offspring larvae across experiments was slightly reduced but this effect was not statistically significant (Supplementary Data Sheet 1—Figure S5B). Similar results were obtained in additional settings in which the food was exposed to micro-organisms carried by naïve flies (Supplementary Data Sheet 1—Figure S5C). Thus, gut bacteria had a significant preventive effect only on the inheritance of the delay in development.

To determine if reintroduction of commensal *Acetobacter* species can prevent the delay in development also in offspring of bacterial depleted flies that have not been exposed to G418, we added specific bacterial species to the food of the F1 larvae that hatched from dechorionated (and sterilized) eggs. Analysis of the rate of larval development in F2 showed that *Colony 1* completely rescued the delay in these offspring of bacterial-depleted flies (Figure 2G). As in the case of parental G418 exposure, non-commensal *A. aceti* had no effect on the delay in F2 (Figure 2H). Additionally, isolated bacteria (*Colony 7*) with 98.9% 16S rRNA similarity to *L. plantarum* (Figure 2B) and *L. plantarum* from another stock (Sharon et al., 2010), led to only partial rescue (Figures 2G,H). Thus, commensal *Acetobacter* species in the F1 parents are necessary for complete prevention of the delay in the F2 offspring. This indicates that lack of these *Acetobacter* species following exposure to G418 is sufficient to account for the heritable delay.

Altogether, the results show that G418 induces a delay in larval development by a direct stress to the host (Figure 1B, Supplementary Data Sheet 1—Figure S3A), but the delay is inherited in the non-exposed offspring because of a transgenerational effect of *Acetobacter* depletion in their parents (Figures 1C,D, 2A,B). This indicates that the induction of the developmental delay and its transgenerational inheritance occur by different mechanisms.

**Figure 3:**
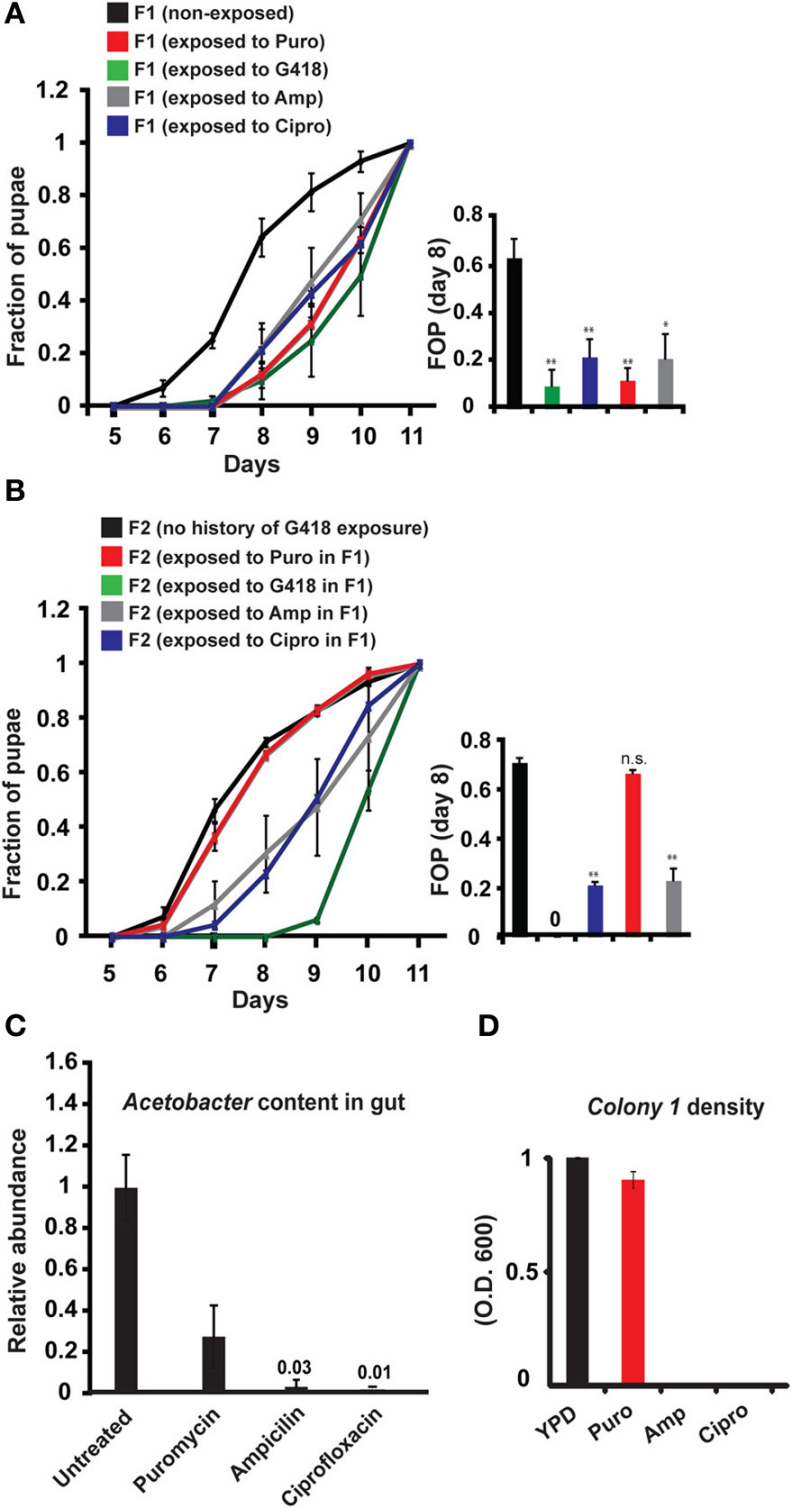
**Generality of involvement of *Acetobacter* species in the inheritance of delayed development. (A)** Kinetic curves of pupation of wild-type F1 *yw* larvae exposed to sub-lethal dosages of four different antibiotics: G418 (100 ug/ml), Ampicillin (100 ug/ml), Puromycin (200 ug/ml), and Ciprofloxacin (300 ug/ml). Mean fraction of pupae ± SE in at least three biological replicates. Inset: Statistics of differences between fractions of pupae (FOP) in day 8. **(B)** Kinetic curves of pupation of non-exposed F2 offspring of F1 flies that were exposed to G418, Ampicillin, Puromycin and Ciprofloxacin. Mean fraction of pupae ± SE in at least 5 vials. Inset: Statistics of differences between fractions of pupae (FOP) in day 8. **(C)** Measured abundance of *Acetobacter* spp. in the gut of third instar larvae exposed to various antibiotics. Mean abundance normalized to non-exposed larvae ± SE in three biological replicates. **(D)** Same as **(C)** for population density of *Colony 1* in YPD media supplemented with these antibiotics. Mean OD_600_ ratio measured after 24 h and normalized to the ratio in YPD alone ± SE in four biological replicates. ^*^p < 0.05, ^**^p < 0.005 (Student's *t*-test).

To evaluate the generality of involvement of *Acetobacter* species in the inheritance of delayed development, we exposed non-transgenic, *yw* flies to other types of antibiotics, supplemented at sub lethal dosages. We established antibiotic-specific dosages that induce a 2–3-day delay in larval development (G418—100 ug/ml, Puromycin—200 ug/ml, Ampicillin—100 ug/ml, and Ciprofloxacin—300 ng/ml). All these exposures to antibiotics induced developmental delay (Figure 3A), which except for the Puromycin case, persisted in subsequent generations without further exposure to the antibiotic (Figure 3B). Since Puromycin stood out as the only antibiotic which did not induce heritable delay, we suspected that it does not sufficiently deplete bacteria which are capable of preventing the inheritance. To test this, we examined the response of *Acetobacter* spp. to exposure with the same concentration of antibiotic. We found that Ampicillin and Ciprofloxacin removed nearly all of the *Acetobacter* spp. from the larval gut and abolished the growth of *Colony 1* in suspension (Figures 3C,D). Exposure to Puromycin, on the other hand, spared a substantial fraction of these bacteria (~1/3 of the *Acetobacter* spp. content of untreated flies; Figure 3C). Additionally, Puromycin had almost no effect on *Colony 1* in suspension (Figure 3D). Thus, substantial *Acetobacter* content could not prevent the delay in Puromycin-exposed flies, but nonetheless prevented the inheritance of the delay in their non-exposed offspring. These results show that the transgenerational effect of *Acetobacter* depletion mediates the inheritance of delayed development in response to additional antibiotics, and that these findings are not limited to transgenic flies.

### *acetobacter*-dependent prevention of the heritable delay is mediated by bacterial riboflavin (vitamin B2)

**Figure 4:**
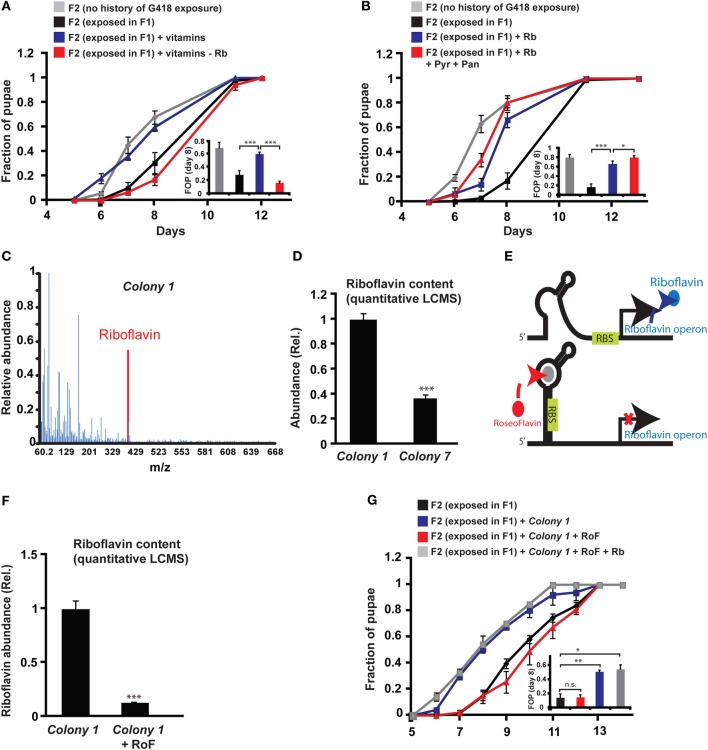
**Riboflavin produced by *Colony 1* is necessary for *Colony 1*-mediated prevention of the inheritance of the delay in development. (A)** Effect of exogenous vitamins on the inheritance of delay in development. G418-exposed F1 *hairy::neoGFP* flies were allowed to lay eggs on G418-free food, supplemented with a full set of six vitamins (Riboflavin, Pyridoxine, Pantothenic acid, Thiamine, Nicotinic acid, Biotin) or with a set from which Riboflavin was excluded. Mean fraction of pupae ± SE in 10 vials. Inset: Statistical analysis of differences between fractions of pupae (FOP) in day 8. **(B)** Same as **(A)** with Riboflavin (Rb) alone (instead of the vitamin pool) or with Riboflavin together with Pyridoxine (Pyr) and Pantothenic acid (Pan). Mean fraction of pupae ± SE in 5 vials. Inset: Statistics of differences between fractions of pupae (FOP) in day 8. **(C)** Mass spectrum of *Colony 1* extract indicating the presence of Riboflavin. Spectrum normalized to the highest peak. **(D)** Quantitative liquid chromatography Mass Spectrometry (q-LCMS) measurements of Riboflavin in extracts of *Colony 1* and *Colony 7*. Mean abundance relative to *Colony 1* ± SE in six biological replicates. **(E)** Schematic diagram of the current model of bacterial Riboflavin riboswitch and its inhibition by Roseoflavin. **(F)** Quantitative LCMS measurements of Riboflavin abundance in *Colony 1* with or without Roseoflavin (RoF). Mean abundance ± SE in three biological replicates. **(G)** Same as **(A)** with Roseoflavin (red) and Roseoflavin together with Riboflavin (blue). Mean fraction of pupae ± SE in five vials. ^*^p < 0.05, ^**^p < 0.005, ^***^p < 0.001 (Student's *t*-test).

Since the bacteria may provide vitamins and nutrients to the larvae, we tested if vitamins could influence the heritability of the delay in development. In particular, we tested the effect of six vitamins that have been shown to be essential additives for germ-free flies reared on axenic food (Sang, 1956). Supplementing all six vitamins to the food prevented the inheritance of the delay in development (Figure 4A), but not the inheritance of the induced expression (Supplementary Data Sheet 1—Figure S6A). To test which of the six vitamins is essential for preventing the inheritance of the delay, we repeated the experiment with one vitamin excluded from each pool. Of the six vitamins tested, Riboflavin (vitamin B2) had the strongest effect on the inheritance. Pools lacking Riboflavin could not prevent the delay in the non-exposed offspring of G418-exposed parents (Figure 4A), indicating that Riboflavin is necessary for the activity of the full vitamin pool. Removal of Pyridoxine (vitamin B6) also compromised the activity of the pool (Supplementary Data Sheet 1—Figure S6B), while the remaining vitamins, Pantothenic acid, Thiamine, Nicotinic acid and Biotin, had no significant effect on the delay in offspring development (Supplementary Data Sheet 1—Figures S6C–F).

Supplementing the food with Riboflavin alone reduced, but did not completely eliminate the delay in development of the non-exposed offspring (Figure 4B). Co-supplementing Riboflavin together with Pyridoxine and Pantothenic acid increased the effect of Riboflavin alone, but only by a small amount (Figure 4B). To test if G418-depleted bacteria produce Riboflavin, we analyzed bacterial extracts of *Colony 1* by Mass Spectrometry. We identified a clear signature of Riboflavin in the extract of *Colony 1* (Figure 4C). Quantitative Liquid Chromatography Mass Spectrometry (q-LCMS) analysis further showed that the amount of Riboflavin produced by *Colony 1* is substantially larger than the amount produced by commensal Lactobacilli from *Colony 7* (Figure 4D). These results suggested that the ability of *Colony 1* to prevent the inheritance of the delay is mediated, at least in part, by production and secretion of Riboflavin. We tested this by supplementing the food with Roseoflavin (RoF), an inhibitor of bacterial Riboflavin synthesis (Lee et al., 2009). Riboflavin is the product of 5 genes encoded by a single polycistronic transcript that is subjected to negative feedback via a riboswitch mechanism; Riboflavin-derived Flavin mononucleotide, FMN, binds to riboswitch aptemers in the transcript, changing the conformation of the transcript into a translationally inactive form, thereby repressing the production of Riboflavin biosynthesis genes (Figure 4E). The FMN analog, Roseoflavin, inhibits Riboflavin production by binding to this riboswitch. We identified a range of Roseoflavin concentrations which did not significantly compromise the growth of *Colony 1* (Supplementary Data Sheet 1—Figure S6G). Nonetheless, supplementing the food with Roseoflavin within this range repressed the production of Riboflavin by 10-fold, as determined by quantitative LCMS (Figure 4F). Consistent with the hypothesized influence of Riboflavin, the inhibition of its synthesis by Roseoflavin eliminated the ability of *Colony 1* to prevent the delay in the offspring (Figure 4G). This outcome was completely reversed by co-supplementing the food with exogenous Riboflavin (Figure 4G), indicating that the effect of Roseoflavin was indeed mediated by its inhibitory influence on the synthesis of bacterial Riboflavin. Altogether, these results show that Riboflavin produced by a commensal *Acetobacter* species interferes with the inheritance of delayed development and inhibition of Riboflavin synthesis in the bacteria compromises the ability of the bacteria to prevent this inheritance.

## Discussion

In this work we investigated the potential involvement of the gut microbiome in the inheritance of environmentally-induced phenotypes. In particular, we tested if G418-mediated disruption of the intact microbiome could account for the inheritance of induced phenotypes in the host. We found that G418 leads to selective changes in the microbial gut composition and that these changes persist in the next generation. This inheritance of microbial disruption raises two potential scenarios which may support inheritance of induced phenotypes. Both scenarios are based on host response to the modifications in the gut bacteria. In the simpler, and more expected scenario, disruption of the gut microbiome modifies host phenotypes within one generation and this response is roughly the same in the following generations (which inherit the changes in the microbiome). This scenario might be expected if the germline is not modified by the disruption of the microbiome. In this case, persistence of the microbial changes creates the phenotypes anew in each generation. In the second potential scenario, the disruption of the microbiome has a different influence on the parents and their offspring. This difference between parents and offspring could suggest an effect of microbial disruption on the parental germline.

Our bacterial removal experiments revealed a clear signature of the second scenario. This was demonstrated by the dramatic difference in pupation time between extracellular bacteria-free parents and their offspring. Our rescue experiments with specific bacterial species further showed that as long as the parents are depleted of their commensal *Acetobacter* spp., the development of their offspring is delayed compared to the development of the parents. Together with the selective depletion of *Acetobacter* spp. by G418, this inter-generational difference accounts for the inheritance of the delay in development in offspring of G418-exposed parents. This scenario of inheritance was unexpected because the induction and inheritance of the delay in development are mediated by different mechanisms. Specifically, we show that the delay in pupation time is induced in the parental generation by the direct influence of G418 on the host, whereas the delay in the offspring generation is caused by the transgenerational influence of *Acetobacter* depletion. Reaching this conclusion requires bacterial depletion and rescue without G418, thus enabling de-coupling of the influence of bacteria from the toxic influence of G418 (Ridley et al., 2013).

The difference between generations with respect to pupation time, clearly indicate that the influence of bacteria in one generation can be different than the influence in the following generation. We hypothesize that this reflects an influence of microbial disruption on the germline. While bacterial-mediated manipulation of the germline has been previously noted for germline-residing endosymbionts (Bourtzis et al., 1996; Werren, 1997; Starr and Cline, 2002; Fast et al., 2011), it has not yet been shown for extracellular gut bacteria. This does not at all negate the diverse influence of gut bacteria on the development and physiology within generation. Indeed, we found that larvae which hatched from dechorionated and sterilized eggs are very sensitive to food quality and, upon reduction in quality, exhibited a considerable delay in development already in the first generation without bacteria (Supplementary Data Sheet 1—Figure S2). Previous studies of gut bacteria in flies further uncovered substantial involvement of the micrbiome in the fly development and homeostasis, including the regulation of larval growth (Shin et al., 2011; Storelli et al., 2011), immune activation (Charroux and Royet, 2012), lifespan determination (Brummel et al., 2004; Ben-Yosef et al., 2008), fecundity (Ben-Yosef et al., 2010), and even mating choice (Sharon et al., 2010). Many past studies, however, assumed that the effect of gut bacteria is the same in each generation. Accordingly, they examined the effect of bacterial removal in flies that had been kept without bacteria for multiple generations prior to the experimental analysis. Under these settings, the transgenerational effect of bacterial removal is influencing both parents and offspring thus, eliminating the ability to determine which part of the response reflects an influence within generation and what part is due to the transgenerational effect. The ability to resolve the different scenarios is further reduced by treatments with antibiotics, which are often used to maintain bacteria-free conditions after dechorionation.

We have previously shown that G418 toxicity induces additional phenotypes beyond the delay in development. These include induced expression of *neoGFP* in gut regions of *hairy::neoGFP* and *drm::neoGFP* larvae, morphological abnormalities in the wings of *Hsp70::neoGFP* flies, reduction in fly size in G418-exposed *drm::neoGFP* larvae, down-regulation of a number of *Polycomb* group genes in the proventriculus of *hairy::neoGFP* larvae, and up-regulation of detoxification (*GstD*) genes in the same region (Stern et al., 2012). Here we show that removal of gut bacteria without exposure to G418 does not induce these other G418 reported phenotypes. This indicates that these phenotypes are caused by a direct effect of the toxic stress of G418 on the fly's tissues, and not by its effect on the fly's microbiome. Additionally, unlike the delay in pupation time, the other heritable phenotypes of G418 exposure (induced expression of *neoGFP* and wing abnormalities) were not observed in offspring of bacterial depleted parents. Thus, the inter-generational difference between parents and offspring did not extend to other heritable phenotypes reported in Stern et al. (2012). Altogether, this indicates that the inheritance of these phenotypes following parental exposure to G418 reflects additional mechanisms beyond those of host–microbe interactions. Recent work in mice also provided evidence for multiple mechanisms supporting the inheritance of different phenotypes in a given experimental setting (Padmanabhan et al., 2013). Having more than one mechanism of transgenerational inheritance in a single experimental setting (e.g., exposure to G418), and in different animals, suggests that non-Mendelian inheritance might be more prevalent than often assumed. Additionally, by uncovering the contribution of microbial disruption to the inheritance of a stress-induced phenotype, our findings expand the set of factors which could support transgenerational inheritance of induced responses.

The transgenerational effect of bacterial removal on pupation time was completely rescued by supplementing the parental food with bacteria from a commensal *Acetobacter* species (*Colony 1*). On the other hand, commensal *Lactobacillus* spp. (*Colony 7*, and *L. plantarum*) led to only partial rescue. The complete rescue by *Acetobacter* species indicates that their absence is sufficient for causing a delay in offspring generations. Additionally, this rescue of the transgenerational effect is consistent with the *Acetobacter*-mediated prevention of the developmental delay in offspring of G418-exposed parents. This prevention was observed in every experimental setting in which G418-free food was exposed to *Acetobacter* spp., including natural forms of contact with bacteria from non-exposed (male or female) parents. Altogether, these results indicate that depletion of *Acetobacter* spp. in both parents is necessary and sufficient for the inheritance of the delay in development. Since the bacterial composition, including *Acetobacter* content, may be influenced by a diverse set of environmental agents (and not just by antibiotics), the microbiome likely mediates multi-generational influences on growth kinetics in response to a variety of natural environmental settings.

The mechanistic basis of bacterial–host interactions in flies has been previously focused on the involvement of Insulin signaling (case of *A. pomorum* Shin et al., 2011) and the TOR pathway (case of *L. plantarum* Storelli et al., 2011). Here we identified Riboflavin as one of the mediators of *Acetobacter*-dependent prevention of the delay in larval development. We found that a commensal *Acetobacter* species produces relatively large amount of Riboflavin. Supplementing the food with exogenous Riboflavin reduced the delay in non-exposed larval offspring of G418-exposed parents. Conversely, co-supplementing the food with *Acetobacter* species and the Riboflavin inhibitor, Roseoflavin, compromised the production of Riboflavin by the *Acetobacter* species and inhibited its ability to prevent the delay in development. This inhibition was relieved by addition of exogenous Riboflavin. Collectively, these results indicate that Riboflavin is necessary for the *Acetobacter*-dependent prevention of the inheritance of the delay in development.

The contribution of Riboflavin to the prevention of the delay in offspring of bacterial depleted parents was also consistent with the smaller rescue ability of commensal *Lactobacillus* spp., *Colony 7*, and *L. plantarum*. Indeed, quantitative LCMS-based analysis of *Colony 7* revealed substantially lower amounts of Riboflavin compared to the commensal *Acetobacter* species (*Colony 1*).

The influence of Riboflavin is also consistent with previous studies which showed that vitamins affect the rate of development of germ-free larvae when the food conditions are also axenic (Tatum, 1939; Sang, 1956). In our settings of non-axenic rich diet, Riboflavin did not influence the growth of non-manipulated flies. Nonetheless, it expedited the development of non-exposed larval offspring of G418-exposed parents. This suggests that the history of exposure to G418 creates an additional requirement beyond that provided normally in the fly's diet.

Deciphering downstream mechanisms of Riboflavin action in host tissue is highly confounded by the broad influence of Riboflavin on many enzymes. Riboflavin is a source of FAD and FMN derivatives serving as cofactors in the oxidative metabolism of carbohydrate, amino acids, and fatty acids. These cofactors are involved in many functions and can have pleiotropic effects on development and physiology (reviewed in Powers, 2003). Similarly to other vitamin-derived cofactors, deficiency in Riboflavin supply (e.g., in the case of bacterial depletion) likely impacts multiple molecular pathways in host tissues. Future work will determine if the effect of Riboflavin deficiency following exposure to G418 is primarily mediated by a single host pathway or, alternatively, by a coordinated action of multiple mechanisms.

Non-Mendelian Transgenerational phenomena in multiple organisms can involve a variety of epigenetic mechanisms (reviewed in Jablonka and Raz, 2009; Daxinger and Whitelaw, 2012; Jablonka, 2012; Lim and Brunet, 2013). In this work we describe a previously unrecognized scenario of transgenerational inheritance mediated by microbial-based modifications in parents which, in turn, affect the development of their progeny. While we demonstrate this in flies, it might be more broadly applicable to many organisms which maintain complex interactions with their commensal bacteria. Indeed, commensal bacteria are an integral part of many, if not all other known animals. These bacteria can be viewed as a “distributed organ” which can be readily affected by the environment on rapid time scales (within and across several generations). The changes in the microbiome might be in species composition as well as in bacterial gene sequence (i.e., following bacterial adaptation to the stressful environment). These changes feedback on host development and physiology, and may be responsible for diverse effects which could have been previously attributed to host-intrinsic factors. The heritability of the changes in the microbiome further provides potential infrastructure for influencing many generations (Bakula, 1969; Rosenberg and Zilber-Rosenberg, 2011). As such, the involvement of commensal bacteria in non-Mendelian, multi-generational responses to the environment might be considerably widespread. Thus, commensal bacteria are major mediators of gene-environment interactions with potential transgenerational and evolutionary implications that await further exploration. The diverse genetic tools available in *D. melanogaster*, together with the relatively low complexity of its microbiome and the ease with which this microbiome can be manipulated, make the fly a powerful model system for this exploration.

## Materials and methods

### *drosophila* stocks

*drm*-*GAL4*, *hairy*-*GAL4, and Hsp70-GAL4* lines were obtained from the Bloomington Stock Center. *yw* line was obtained from the lab of Dr. Eli Arama (Weizmann Institute of Science), *Canton-S* and *Oregon-R* lines were obtained from the lab of Prof. Adi Salzberg (The technion). The UAS-*neoGFP* line was generated as described in Stern et al. (2012).

### Food preparation

Standard cornmeal food (Bloomington Stock Center recipe, http://flystocks.bio.indiana.edu/Fly_Work/media-recipes/molassesfood.htm) was heated to ~60°C and mixed (1:100 in volume) with antibiotics to reach the desired final concentration. For the experiments involving the transgeneic, *hairy::neoGFP*, *drm::neoGFP* and *Hsp70::neoGFP* lines we used G418 at final concentration of 400 μg/ml. For wild-type strains (*yw, Canton-S, Oregon-R*), final concentrations of antibiotics were as follows: G418 (100 μg/ml), Ampicillin (100 μg/ml), Puromycin (200 μg/ml), and Ciprofloxacin (300 μg/ml). The food was divided into standard 25 × 95 mm Drosophila vials (cat# 51–0500, Biologix, USA). Vials with 10 ml of food were left overnight at room temperature and stored at 4°C for up to 2 weeks prior to usage. For non-exposed conditions, the food was prepared in the same way but without the antibiotics.

### Measuring duration of larval development

Duration of larval development was measured by counting the number of pupae in each vial daily. The integrated number of pupae formed prior to each inspection time was normalized to the total number of pupae formed in the vial at the end of the experiment.

### Isolation and growth of commensal bacteria

Ten male flies were shaken in 1 ml of PBS buffer at room temperature for 30 min. 100 ml of this fluid were serially diluted, spread on YPD agar plates and colonies were grown at room temperature for 3 days. Several colonies were picked, underwent three additional rounds of isolation, grown overnight at 30°C in liquid YPD and stocked in 35% glycerol at −80°C.

### Transgenerational experiments

Three males and two females were crossed and allowed to lay eggs for 3 days in vials with or without G418 (or other antibiotics). Unless specifically indicated, all F1 experiments with transgenic flies were done using lines heterozygous for the *GAL4* driver and the UAS-*neoGFP* transgenes, as described in Stern et al. (2012). F1 flies developed from these eggs were collected after 19–20 days from the start of the experiment (4–7 day old adults) and the same number of males and females were crossed again in vials without antibiotics. For subsequent generations in experiments involving the transgenic lines, only fluorescent adults were crossed (i.e., flies carrying at least one *GAL4* and one UAS-*neoGFP* transgenes).

### Transgenerational experiments with prior removal of bacteria from flies

Dechorionated and sterilized embryos were transferred to vials with or without G418. For analyses of F2 and F3 bacterial depleted flies, F1 flies developed from these eggs were collected after 19–20 days from the start of the experiment (4–7 day old adults) and the same number of males and females were crossed again in vials without G418.

### Statistical analyses

All statistical tests were performed using the MATLAB software (MathWorks). One-sided student's *t*-test was used for evaluating the statistical significance of mean values. Analyses of functional enrichments in groups of up- and down-regulated genes in the microarray experiments were done using the DAVID web tool (http://david.abcc.ncifcrf.gov/, Huang da et al., 2009a,b).

### Detection and quantification of *neoGFP* expression

Quantification of *neoGFP* intensity in the proventriculus: For each proventriculus of an individual third instar larva, an image was taken using a fluorescent stereoscope (Leica MZ16F) with constant imaging parameters. Image analysis for measuring the induction of *neoGFP* in the proventriculi of *hairy::neoGFP* larvae was performed as follows: GFP intensity was measured in the anterior half of the proventriculus by computing the average pixel intensity of GFP. Then, for each image the background auto-fluorescence of the tissue (evaluated in the dark area in the middle of the proventriculus) was subtracted. Image analysis and computation was performed using a custom MATLAB (MathWorks) script.

Quantification of *neoGFP* intensity of expression in the midgut: images were taken from each larva as described above for the proventriculus tissue. Quantification of *neoGFP* levels in the midgut of *drm::neoGFP* larvae was performed by calculating the average pixels intensity in the region of the midgut with *GFP* intensity above background. Measurements from non-exposed larvae were performed in a midgut tissue of about the same size and location. Average intensity was corrected for auto-fluorescence in the midgut (primarily due to food inside the gut) by subtracting the average intensity measured in an adjacent (non-induced) midgut area.

### Detection and quantifications of pupa and adult phenotypes

Images of adult males or females were taken using a fluorescent stereoscope (Leica MZ16F). Length of adult *drm::neoGFP* flies was calculated by measuring (using the NIS-Elements software, Nikon) the number of pixels along a line drawn from head to genital of each fly. The number of pixels was then converted to millimeters.

Adult *Hsp70::neoGFP* flies with wing abnormalities were scored by eye.

### Conditioning experiments

Conditioning experiments with flies were performed by insertion of 5 adult male flies into a regular fly vial. After 24 h, these flies were discarded and replaced by flies that were used for mating. Conditioning with wash fluid was performed by washing 10 adult flies in 1 ml of PBS for 30 min. 100 ml of the wash fluid was applied to the top of the fly food. After 24 h these vials were used for the mating experiments.

### Analysis of 16S rRNA sequence of *colony 1* and *colony 7*

The 16S gene was PCR-amplified from bacterial DNA using universal primers 8F (AGAGTTTGATCCTGGCTCAG) and 1492R (GGTTACCTTGTTACGACTT) (Weisburg et al., 1991). The PCR product was cloned into PGMT plasmid, sequenced and compared to the 16S database (Green Genes database http://greengenes.lbl.gov/) using the BLAST algorithm. The resulted closest matches—*A. aceti* (AJ419840.1), *A. pomorum* (AJ001632.1), *A. tropicalis* (AJ419842.1), and *A. cibinongensis* (AB052711.1), along with the previously reported commensal bacteria *L. plantarum* (JQ411248.1), *L. brevis* (JQ236623.1), *L. fructivorans* (AB680532.1), and Wolbachia (DQ412083.1) were used for multiple alignment using the T-Coffee web resource.

### Bacterial strains from commercial stocks

*A. aceti*, *A. tropicalis, A.pomorum, and A.cibinongensis* were purchased from the German Collection of Microorganisms and Cell Cultures (DSMZ, http://old.dsmz.de/identification/main.php?contentleft_id=2) and were grown in YPD or LB media.

### Sample preparation for scanning EM

Bacteria were grown to mid-log in YPD medium at 37°C and were fixed with 2.5% gluteraldhyde and 2.5% PFA in 0.2 M cacodylate buffer. The cells were attached to PLL (poly-L-lysine) coated silica chip, dehydrated in an ethanol series (50, 70, 96, and 100%) and critical-point dried using BALTEC dryer. The dry samples were coated with gold-palladium for 4 min and visualized in a high-resolution Ultra 55 SEM (Zeiss).

### Sensitivity of *colony 1* to different antibiotics

A single colony from the *Colony 1* stock was isolated from YPD plates and grown to OD_600_ = 1 in YPD at 30°C with continuous shaking. 10 μl of this were inoculated into 2 ml of YPD, and different concentrations of antibiotics were added in triplicates. OD_600_ was measured after 24 h of incubation at 30°C. OD measurements were normalized to antibiotic-free sample.

### Bacterial removal by egg dechorionation

Flies were crossed and allowed to lay eggs for 2–4 h. Eggs were collected and dechorionated for 2 min in 2.7% sodium hypochlorite (2-fold diluted bleach), washed twice in 70% ethanol and then twice with sterile distilled water as previously described (Brummel et al., 2004).

### Bacterial reintroduction experiments

For the bacterial reintroduction experiments we used bacteria from single colonies (either *Colony 1* or *Colony 7*) isolated from naive flies, a strain of commensal *L. plantarum* obtained from the Rosenberg lab (Tel-Aviv University), and bacteria from DSMZ collection (*A. aceti, A. pomorum*, *A. tropicalis*, and *A. cibinongensis*). The bacteria were grown (with shaking) overnight to an OD_600_ of 1–2 at 30°C in 3 ml YPD. Bacteria were diluted to OD 0.1 in YPD, and 100 μl were added to each vial before the transfer of untreated or dechorionated eggs to these vials.

For analysis of the F2 generation, 5–10 days old adult flies were collected from these vials, and 3 males and 2 females were crossed in a new (untreated) vial for 2 days.

### Supplementing fly food with vitamins and roseoflavin

Standard cornmeal food (Bloomington Stock Center recipe, http://flystocks.bio.indiana.edu/Fly_Work/media-recipes/molassesfood.htm) was heated to ~60°C and mixed with Thiamine (2·10^−3^ mg/ml final), Riboflavin (1·10^−2^ mg/ml), Nicotinic Acid (1.2·10^−2^ mg/ml), Calcium Pantothenate (1.6·10^−2^ mg/ml), Pyridoxine (2.5·10^−3^ mg/ml), Biotin (1.6·10^−4^ mg/ml), Roseoflavin (100 μM), or combinations thereof dissolved in water. The mix was split to standard 25 × 95 mm *Drosophila* vials (cat# 51–0500, Biologix, USA). Vials with 10 ml of mixed food were left overnight at room temperature before storage at 4°C for up to 2 weeks prior to usage.

### mRNA extraction and analysis

Proventriculi were dissected from ~50 *hairy::neoGFP* third instar larvae for each condition, and were kept during dissection in ice cold PBS. Total RNA was extracted and purified using the RNeasy MinElute Cleanup Kit (QIAGEN). Genome-wide mRNA expression levels in each sample were measured using Affymetrix drosophila 2.0 array using standard Affymetrix protocols. Arrays were normalized with the RMA algorithm using the Expression Console software (Affymetrix).

Results for specific genes were verified using real-time quantitative PCR as follows: mRNA was converted to cDNA using high-capacity Reverse Transcription kit (Ambion). Transcript levels were measured using real-time qPCR on a 7900HT Fast Real-Time PCR Machine using Power SYBR green PCR master mix (Applied Biosystems). Specific primers used in this study are listed below:

**Table:** 

**Gene**	**F primer**	**R primer**
*Act5C*	CCCTCGTTCTTGGGAATGG	CGGTGTTGGCATACAGATCCT
*Pc*	AAATCATCCAAAAGCGCGTTA	CCGGTTCCCAGGTGTTGTAG
*ph-p*	AATTTTGGCCATGACCTCGAT	ACAGCGGTGCTTGTCACAGA
*Scm*	GTTTGCCCTGGAAGGAGATGT	TCATCCTTCATTCGCATTGG
pho	ACGGTCCTCGAGTCCATGTT	CACCGGTGTGAACCAACTGA

### Mass spectrometry analysis

#### Mass spectra analysis of colony 1

*Colony 1* was cultured overnight (with shaking) at 30°C in 100 ml YPD to OD_600_ between1.5 and 2. Bacteria were centrifuged at 4°C for 15 min at 4000 g and washed twice with 20 ml of water at the same conditions. The bacteria were re-suspended in 10 ml of water and sonicated on ice using microtip at 40% power (30 s ON followed by 50 s OFF for a total of 15 min) on a Sonics Vibra-Cell machine (Sonics and Materials, Inc.). The solution was then centrifuged (20,000 g) at 4°C for 20 min and the supernatant was passed through 0.22 micron filter. Pure vitamins [Riboflavin—Sigma-Aldrich R4500–25; Calcium D−(+)-pantothenate—Santa-Cruz SC-202515; Pyridoxine hydrochloride—Sigma-Aldrich P9755–25G] were reconstituted from powder to serve as references for the bacterial samples. All samples were analyzed on an Electron Spray machine in the Mass Spectrometry core facility unit at the Weizmann Institute of Science, Rehovoet, Israel.

#### Quantitative measurements of riboflavin content

Bacteria were cultured (with shaking) overnight to OD_600_ between 1 and 2 at 30°C in 20 ml YPD. For experiments with Riboflavin inhibition, Roseoflavin (0215425225, ENCO) was added to the YPD to a final concentration of 100 μM and the bacteria were allowed to grow. It was then diluted to OD_600_ 1, and 10 ml were centrifuged at 4°C for 15 min at 4000 g and washed twice with 10 ml of ice-cold DDW. The bacteria were re-suspended in 1 ml of methanol and sonicated on ice using “Bioruptor® Standard sonication device” on high setting (30 s ON followed by 30 s OFF for a total of 30 min). The solution was then centrifuged (20,000 g) at 4°C for 20 min and the supernatant was passed through 0.22 micron filter. It was completely evaporated with Nitrogen gas and re-suspended in 100 μl methanol. Pure vitamins (Riboflavin—R4500–25 and Thiamine hydrochloride—T4625–25G, Sigma-Aldrich) were reconstituted from powder to concentrations of 50 μg/ml to serve as references for the bacterial samples. Three biological repeats of each sample were subjected to LC-MS analysis.

Analysis was performed using an Ultra Performance Liquid Chromatography—Mass Spectrometry (UPLC)-MS/MS triple quadrupole instrument (Xevo TQ MS, Waters) equipped with an electrospray ion source and operated in positive ion mode. MassLynx and TargetLynx software (v.4.1, Waters) were applied for the acquisition and analysis of data. Chromatographic separation was done on a 100 × 2.1-mm inside diameter, 1.7-μm UPLC BEH C18 column (Acquity, Waters) with mobile phases A (10 mM ammonium formate buffer, pH = 6.26) and B (acetonitrile) at a flow rate of 0.3 ml/min and column temperature 40°C. A gradient was as follows: 0–4 min linear gradient from 0 till 100% B, 4.0–4.5 min hold at 100% B, 4.5–5.0 min return to 0% B and equilibration at 0% B for 2 min. Injection volume of 5 μL was used.

For MS, argon was used as the collision gas with flow 0.22 ml/min. Cone voltage was 40 V, the capillary was set to 1.15 kV, source temperature—150°C, desolvation temperature—550°C, desolvation gas flow—800 L/min, cone gas flow—50 L/min. Riboflavin was detected using multiple reaction monitoring (MRM) applying the following parameters: transition 377.2 > 243.1 (collision energy—22 eV, was used for quantification), transition 377.2 > 198.1 (collision energy—36 eV).

### Bacterial DNA-seq experiments

Ten guts of *hairy*::*neoGFP* third instar larvae that were developed in vials with or without G418, were dissected from each sample and pooled together. Bacterial DNA was extracted using the “chemagic DNA bacteria Kit” (Chemagen). The sequencing of the 16S rDNA was performed as described by Amir et al. (2014). Briefly, DNA was PCR amplified using six pairs of non-overalpping 16S primers. Each pair amplified relatively short (~200 bp) region that is conserved among a subset of known bacteria. Altogether, the six regions amplify over 90% of the Greengenes database (version dated 2010), having an amplicon of approximately 1200 base pairs in total. Additionally, the primers included Illumina sequencing barcodes and therefore the library preparation stage was not necessary. PCR products were cleaned (Promega, Fitchburg, WI) and DNA concentration was measured using Qubit (Life Technologies). All samples were pooled in equimolar ratios and sequenced on a single lane of an Illumina HiSeq2000 sequencer using 100 nt paired-end reads. The number of reads per sample following quality filtering was about 20·10^6^.

Microbial profiling based on the 16S sequences was performed using a novel framework which allows massively parallel sequencing (MPS) of a large genomic region, thus increasing the resulting phylogenetic resolution while reducing the number of false positively detected bacteria. Detailed description of the microbial reconstruction methodology is provided by Amir et al. (2014). In brief, we formulated a statistical model for generating the observed reads given a pre-defined database of about 450,000 known 16S sequences (Greengenes, version dated 2010), and solved an optimization problem whose output is the identity and frequency of each sequence. Each of the millions of reads measured by MPS, together with all “absent” reads that were not found, set constraints which enable “zooming in” on the correct species present in the mixture. Since reads are much shorter than the amplified region and contain errors, they are often shared by many bacterial sequences in the database. Nonetheless, each read provides evidence in support of the existence of the “correct” bacteria in a probabilistic way. The method integrates the statistical evidence from all reads to infer the frequency of each sequence in the database.

### Measuring bacterial content by PCR

For primer specificity tests, we used defined bacteria obtained from the DSMZ stock collection. Colonies were isolated and grown over-night in YPD medium. Wolbachia was omitted from these tests due to lack of culturing conditions. To measure the levels of bacteria in the gut, 7–10 third instar larvae were collected. The gut of the larvae was dissected and pooled. Bacterial genomic DNA was extracted using “chemagic DNA Bacteria” kit (Chemagen) and 5 ng of purified DNA was used per qPCR reaction. All reactions were performed in triplicates on a qPCR machine (Applied Biosystems 7900HT Fast Real-Time PCR System, Life Technologies Corporation) using SYBRGreen in 384 well-plates. The species-specific primers used are: aceto_rt_1_f (TAG TGG CGG ACG GGT GAG TA), aceto_rt_1_r (AAT CAA ACG CAG GCT CCT CC), lacto_rt_2_f (AGG TAA CGG CTC ACC ATG GC), lacto_rt_2_r (ATT CCC TAC TGC TGC CTC CC), wolb_rt_2_f (CAA TGG TGG CTA CAA TGG GC), wolb_rt_2_r (GTA TTC ACC GTG GCG TGC TG). DNA content of the Drosophila *Actin* gene was used to normalize the bacterial content. *Actin* primers used: dros_rt_1_f (GGA AAC CAC GCA AAT TCT CAG T), dros_rt_1_r (CGA CAA CCA GAG CAG CAA CTT).

## Conflict of interest statement

The authors declare that the research was conducted in the absence of any commercial or financial relationships that could be construed as a potential conflict of interest.

## Supplementary material

Supplementary Data Sheet 1 **Supplementary Figures S1–S6 (including captions)**.

Supplementary Data Sheet 2 **Summary of 16S deep-sequencing results of gut samples of G418 exposed and non-exposed larvae**. Samples were extracted from third instar *hairy::neoGFP* larvae. Data is represented as the normalized percent of reads mapped to a Greengenes Database annotation (for bacterial abundance >1%). The 16S ribosomal RNA sequence of each Greengenes annotation was associated with known bacteria by calculating the respective sequence homology using RDP classification (http://rdp.cme.msu.edu/tutorials/classifier/RDPtutorial_RDP-CLASSIFIER.html).

Supplementary Data Sheet 3 **Summary of microarray analyses of mRNA profiles in the proventriculus of bacterial depleted and untreated flies**. mRNA was extracted from the proventriculi of *hairy::neoGFP* third instar larvae that hatched from dechorionated and sterilized eggs or from untreated eggs. Genome-wide profiles were analyzed using affymetrix microarrays. Data represented as log2 fold-change difference between bacterial depleted and untreated larvae in two biological replicates.

Supplementary Data Sheet 4 **Functional enrichments in sets of proventriculus genes which respond to removal of extracellular bacteria**. Analyses of functional enrichments in groups of up- and down-regulated genes (>1.5-fold) performed using the DAVID web tool (http://david.abcc.ncifcrf.gov/).
