# Supplementary material for: Delayed development induced by toxicity to the host can be inherited by a bacterial-dependent, transgenerational effect
Source: Front Genet. 2014 Feb 25;5:27. doi: 10.3389/fgene.2014.00027 (PMC3933808; doi:10.3389/fgene.2014.00027)
Supplement: Supplementary Data Sheet 1 — Supplementary Figures S1–S6 (including captions). [file DataSheet1.PDF]

Supplementary Information

Figure S1

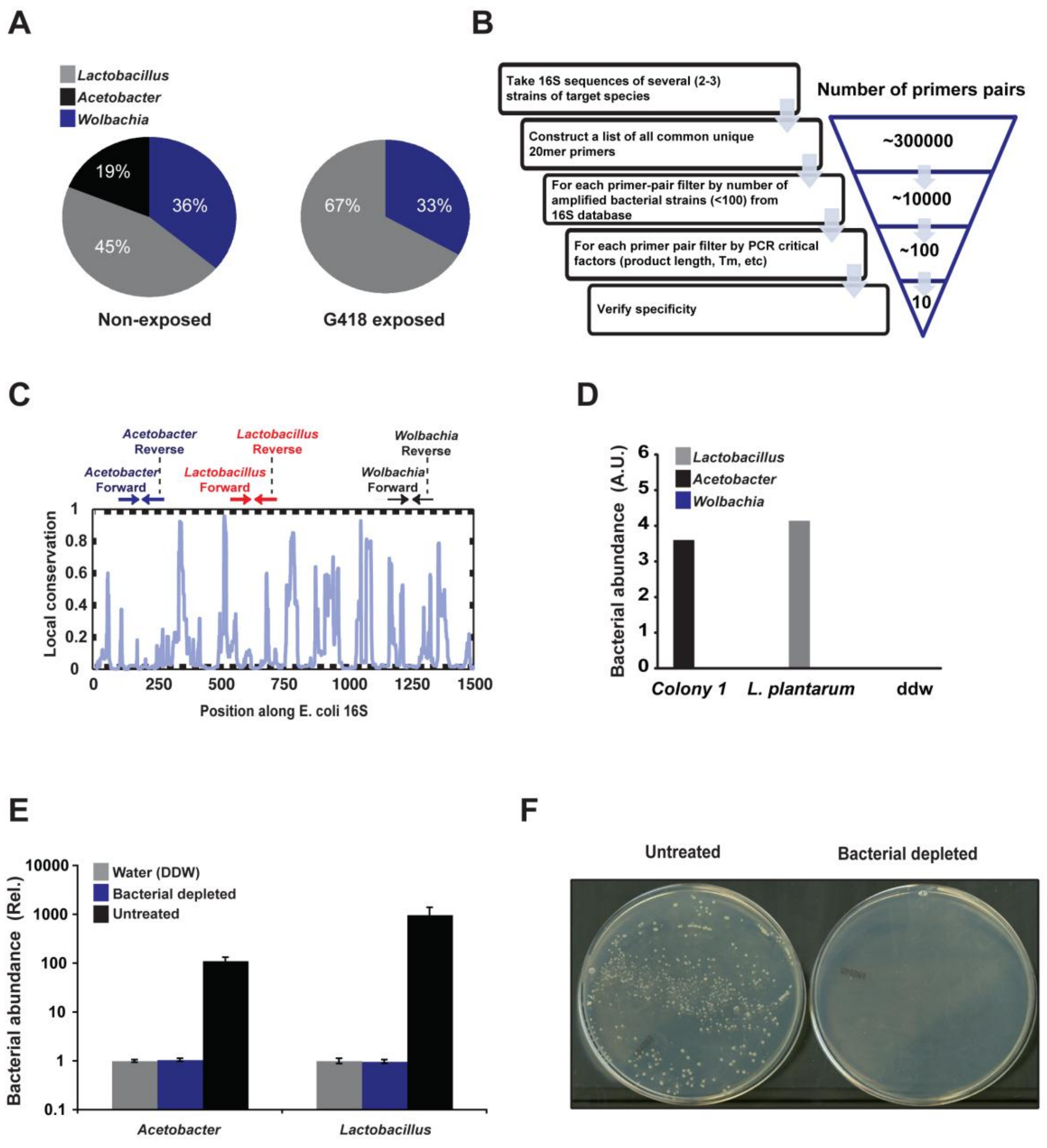

Figure S1: Evaluating changes in the composition of the commensal microbiome
(A) Composition of commensal bacteria and its modification by larval exposure to G418 as determined by deep sequencing of 16S rRNA gene sequences extracted from the gut of 3rd instar, F1 *hairy::neoGFP* larvae. **(B)** Schematics of the design of qPCR probes selective, respectively, for consensus 16S sequences of *Acetobacter*, *Lactobacill*us and *Wolbachia spp.* **(C)** Position of the qualifying qPCR probes (colored arrows), indicated with respect to the phylogenetically conserved regions along the 16S rRNA gene sequence of E. coli. **(D)** Verifying the specificity of the qualifying qPCR probes for detection of commensal *Acetobacter* and *Lactobacillus* species in defined samples (*Colony 1* and *L. plantarum*, respectively). X-axis labels designate the bacterial samples used in each analysis. Estimated relative abundance of

bacteria represented as: 2^-(qPCR cycles)*1000**.** **(E)** Relative abundance of *Acetobacter* and *Lactobcillus* species in the gut of 3rd instar larvae developed from dechorionated and sterilized eggs (GF) or from untreated larvae. Note the complete absence of *Acetobacter* and *Lactobcillus* species in larvae from dechorionated eggs (indistinguishable from sterilized double distilled water). Mean ± SE based on 3 biological replicates. **(F)** Representative images demonstrating, respectively, presence and absence of bacteria in fluid that was incubated with flies developed from untreated (left) and dechorionated and sterilized eggs (right). The fluid was applied to LB plates, under conditions permissive for *Acetobacter* and *Lactobacillus* growth.

Figure S2

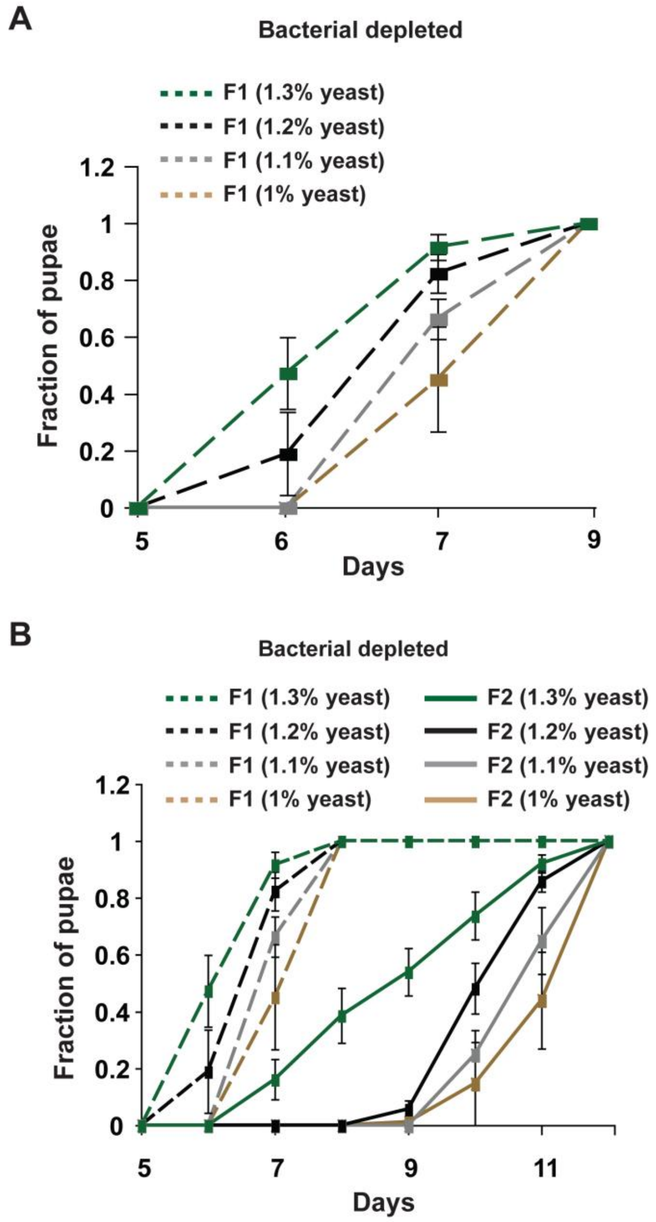

Figure S2: Effect of yeast extract concentration on the rate of development of bacterial-depleted flies
(A) Fraction of pupae formed over time in bacterial-depleted F1 flies (dechorionated and sterilized as embryos) on food with different concentrations of yeast extract (1%-1.3%). Mean fraction of pupae ± SE in 4 vials. **(B)** Same as (A) for fraction of pupa formed over time in F1 and F2, with dechorionation and sterilization in F1. Mean fraction of pupae ± SE in 4 vials.

Figure S3

A

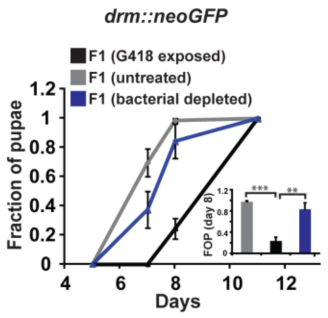

B

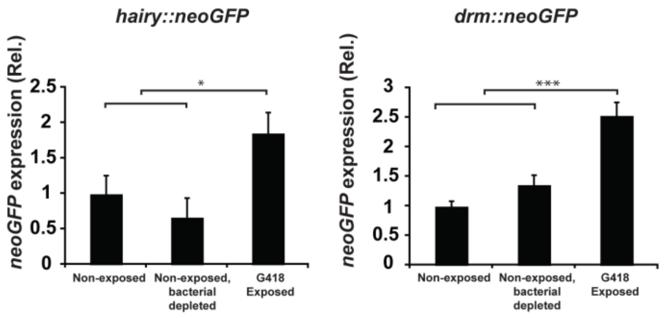

C

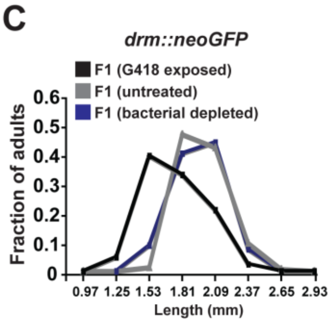

D

Hsp70::neoGFP

Sample	Percent (abnormal wings)	Number of flies
Untreated	0%	322
G418 exposed	2.6%	162
Bacterial depleted	0%	269

E

	# Genes induced	# Genes repressed
G418 exposure	295	526
Bacterial depletion	69	68
Overlap (# of genes)	23	30
Overlap (%)	8%	6%

F

GO term	Bacterial depleted enrichment	G418 exposure enrichment
Phospholipase activity	$P<10^{-8}$	Not enriched
Polysaccharide metabolism	$P<10^{-4}$	Not enriched
Oxidation reduction	Not enriched	$P<10^{-7}$
Glutathione S-transferase	Not enriched	$P<10^{-7}$
Response to bacterium	$P<10^{-4}$	Not enriched
Cell morphogenesis	Not enriched	$P<10^{-16}$
Phosphoprotein	Not enriched	$P<10^{-11}$
Chromosome organization	Not enriched	$P<10^{-6}$
Mitosis	Not enriched	$P<10^{-6}$

G

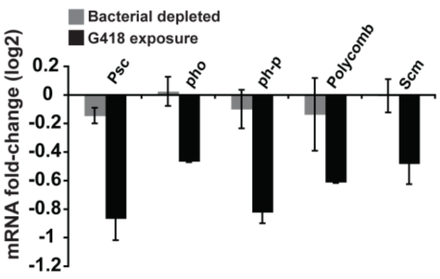

H

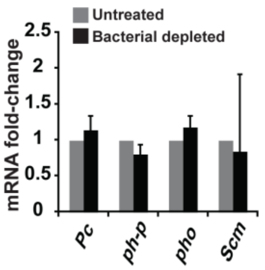

I

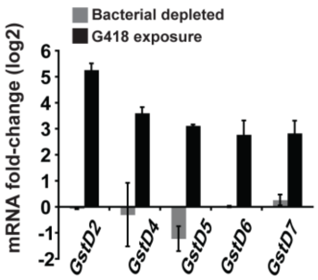

J

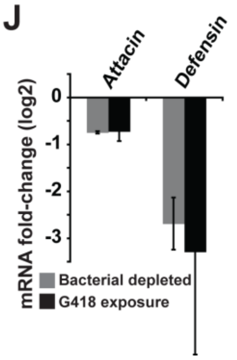

Figure S3: Removal of *Acetobacter* and *Lactobacillus spp.* does not phenocopy the G418-induced phenotypes

(A) Effect of egg dechorionation and sterilization ('Bacterial depleted') on the kinetics of pupation of *drm::neoGFP* larvae in the same generation. Note the small effect compared to the large delay following exposure to G418. Mean fraction of

pupae ± SE in 5 vials. Inset: Statistical analysis of differences between fractions of pupae (FOP) in day 8. **(B)** Left: Expression of *neoGFP* in the proventriculi of 3rd instar F1 *hairy::neoGFP* larvae. Mean GFP intensity ± SE in the proventriculi of G418-exposed larvae (n=42), larvae that hatched from dechorionated and sterilized eggs (n=15), or from intact eggs (n=46). Right: same as (left) for *drm::neoGFP* larvae. n=49, n=41, and n=53, respectively. **(C)** Length distributions of non-exposed adult F1 *drm::neoGFP* flies with intact bacteria (n=157), flies developed from dechorionated and sterilized eggs (n=80) and flies developed from G418 exposed larvae (n=125). **(D)** Percentages of adult F1 *Hsp70::neoGFP* flies exhibiting abnormal wings after: Larval exposure to G418 (n=162), removal of bacteria by egg dechorionation and sterilization (n=269), and without any treatment (322). **(E)** Numbers of genes induced or repressed over 1.5-fold in the proventriculi of bacterial-depleted and G418-exposed 3rd instar *hairy::neoGFP* larvae. Based on microarray data in 2 biological replicates for each condition. **(F)** Examples for enrichments of functional (GO) annotations in the sets of induced and repressed genes referred to in (E). Red and green correspond to induced and repressed sets, respectively. **(G,H)** Microarray-based (G) and qPCR-based (H) measurements of mRNA fold-change of *Polycomb* genes in the proventriculus of 3rd instar F1 *hairy::neoGFP* larvae that were exposed to G418 [14] and in non-exposed larvae developed from dechorionated and sterilized eggs. **(I,J)** Same as (G) for GstD genes (I) and antimicrobial genes (J). * $p < 0.05$, ** $p < 0.005$, *** $p < 0.001$ (Student's t-test).

Figure S4

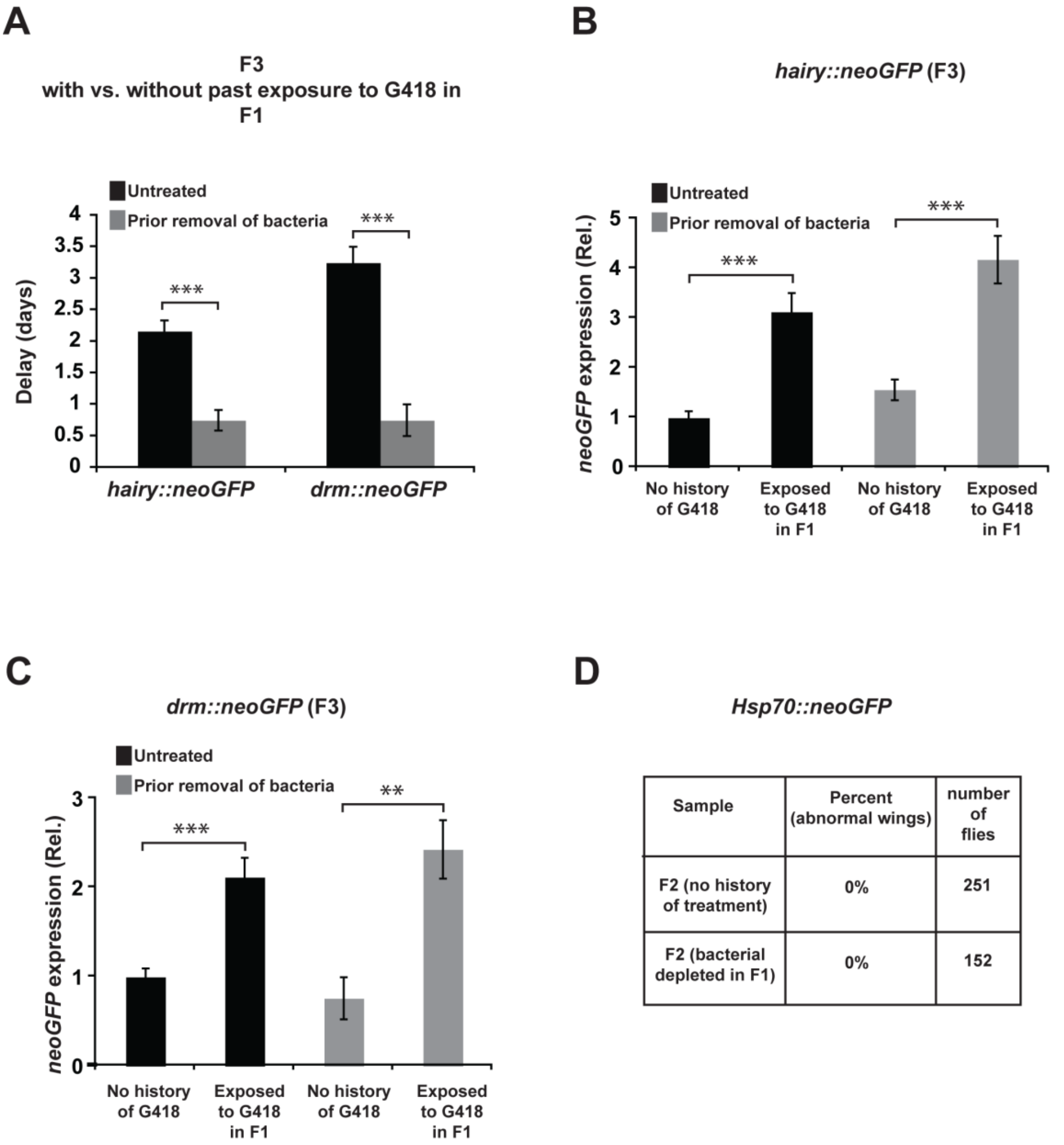

Sample	Percent (abnormal wings)	number of flies
F2 (no history of treatment)	0%	251
F2 (bacterial depleted in F1)	0%	152

Figure S4: The transgenerational impact of bacterial removal is responsible for the inheritance of the delay but not for the inheritance of the other phenotypes
(A-C) Removal of extracellular bacteria prior to G418 exposure affects the inheritance of the delay in development but not the inheritance of induced *neoGFP* expression. *hairy::neoGFP* and *drm::neoGFP* larvae from dechorionated and sterilized eggs were exposed or non-exposed to 400ug/ml of G418 in F1. **(A)** Difference in developmental time in F3 larval offspring compared to offspring lacking ancestral history of G418 exposure. Note the strong effect of bacterial depletion on the inheritance of the delay. Mean ± SE in at least 4 biological replicates. **(B)** Effect of bacterial removal (prior to G418 treatment) on the heritability of the elevated expression of *neoGFP* in 3rd instar F3 *hairy::neoGFP* larvae. Mean GFP intensity ± SE in the proventriculi of larvae with (n=26) or without (n=30) exposure to G418 in F1, and for bacterial depleted larvae with (n=21) or without (n=15) exposure in F1.

(C) Same as (B) for *drm::neoGFP* larvae. Mean GFP intensity ± SE in the midgut of larvae with (n=29) or without (n=21) a history of exposure to G418 in F1, and for bacterial depleted larvae with (n=25) or without (n=13) a history of exposure in F1. **(D)** Fraction of flies with wing abnormalities in F2 offspring of *Hsp70::neoGFP* flies developed from bacterial depleted or untreated parents. ** $p < 0.005$,*** $p < 0.001$ (Student's t-test).

Figure S5

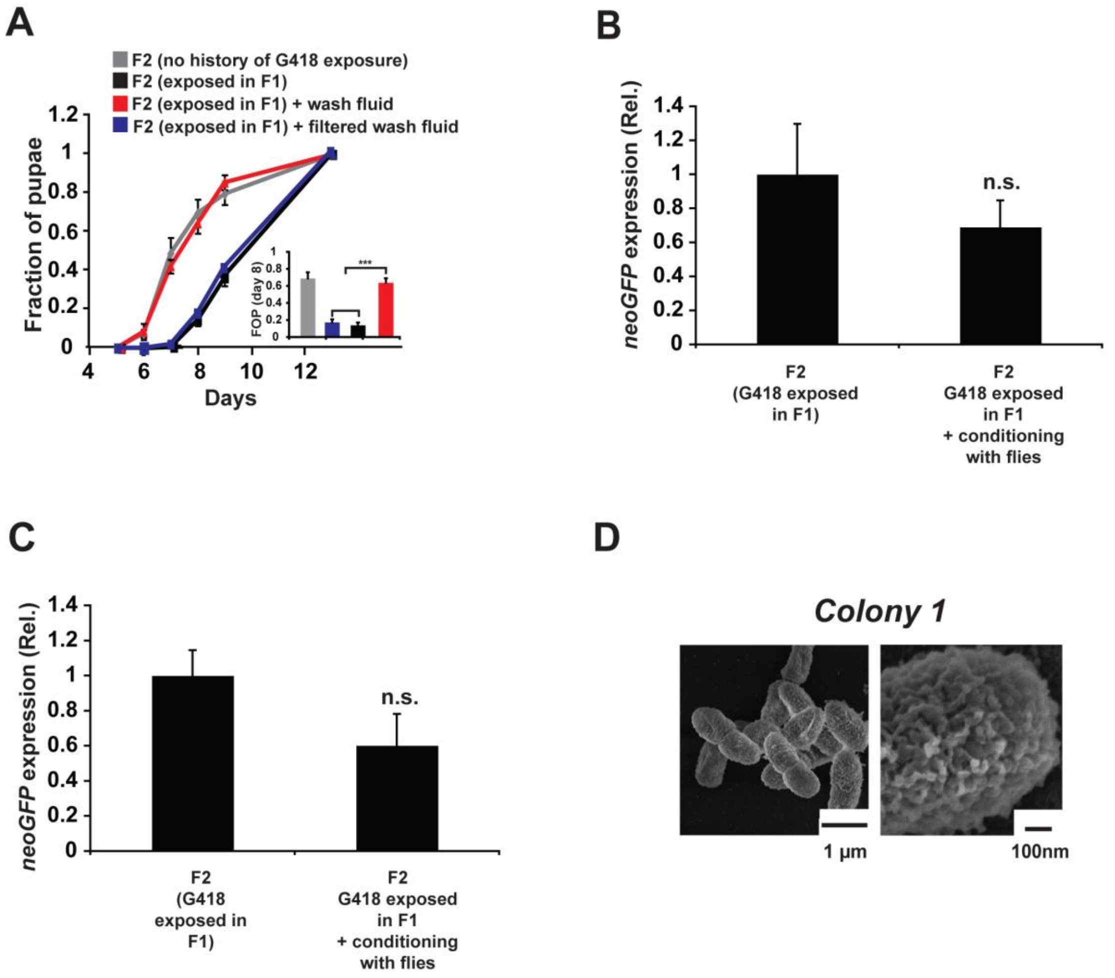

Figure S5: Gut bacteria reproducibly prevent the inheritance of the delay but not the inheritance of induced *neoGFP* expression

(A) Non-exposed offspring of G418 exposed parents were developed in vials supplemented with PBS that was pre-incubated with flies with no history of ancestral exposure to G418 ('wash fluid'). Prior to supplementation, the 'wash fluid' was either filtered to remove microorganisms, or kept intact. Only the non-filtered wash fluid was able to prevent the inheritance of the delay. Mean fraction of pupae ± SE in at least 4 vials. Inset: Statistics of differences between fractions of pupae (FOP) in day 8**.** **(B)** Food supplementation with *Colony 1* had non-significant effect on the inheritance of induced *neoGFP* expression. Mean GFP intensity ± SE of 6 biological replicates. **(C)** Same as (B) for conditioning experiments in which the offspring were developed on food that was temporarily exposed to flies with no history of ancestral exposure to G418. Mean GFP intensity ± SE of 3 biological replicates. **(D)** Electron micrographs of *Colony 1*. * $p < 0.05$, ** $p < 0.005$,*** $p < 0.001$ (Student's t-test).

Figure S6

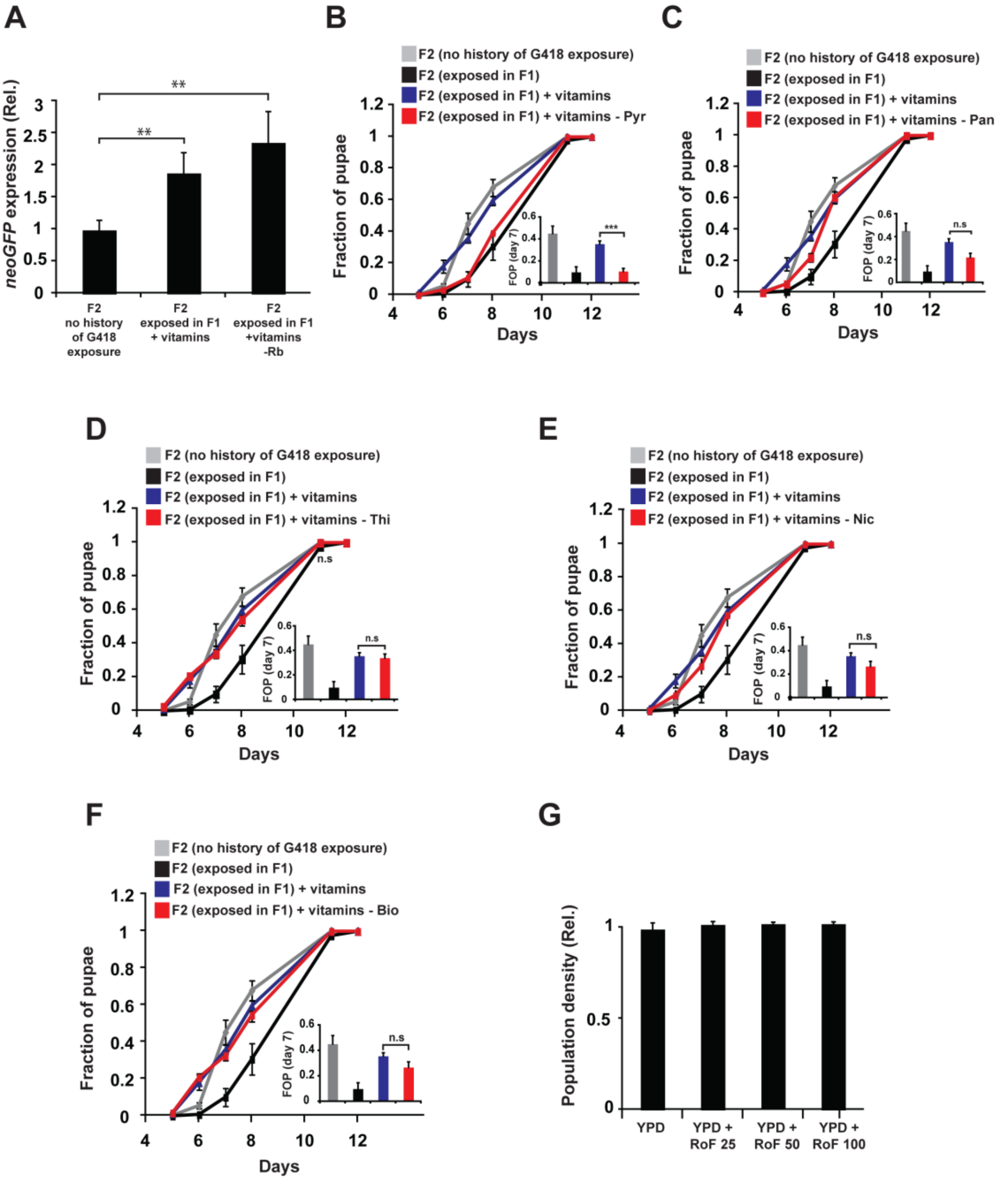

Figure S6: Effects of different vitamin pools on the inheritance of delayed development

hairy::neoGFP flies were reared in vials supplemented with a set of 6 vitamins (Riboflavin (Rb), Pyridoxine (Pyr), Pantothenic acid (Pan), Thiamine (Thi), Nicotinic acid (Nic), and Biotin (Bio)) or with a set in which one of the vitamins was excluded (Riboflavin (A), Pyridoxine (B), Pantothenic acid (C), Thiamine (D), Nicotinic acid (E), and Biotin, Bio (F)). **(A)** The pool of supplemented vitamins (with or without Riboflavin) did not prevent the inheritance of induced gene-expression in the proventriculus of 3rd instar F2 *hairy::neoGFP* larvae. Mean GFP intensity ± SE in the proventriculi measured in the following cases: Non-exposed F2 offspring of flies with no history of G418 exposure (n=34), non-exposed F2 offspring of G418-exposed flies supplemented with the full 6-vitamin pool (n=26) or with the vitamin pool excluding

Riboflavin (n=21). **(B-F)** Kinetic curves of pupation in F2, with and without supplementation of all 6 vitamins or various pools with 5 vitamins. Mean fraction of pupae ± SE in 10 vials. Insets: Statistical analysis of differences between fractions of pupae (FOP) in day 7**. (G)** Population density of *Colony 1* following 24h of growth in YPD supplemented with 25, 50 and 100μM of Roseoflavin (RoF)**.** Mean ± SE in 4 biological replicates. ** $p < 0.005$, *** $p < 0.001$ (Student's t-test).
